# Genetic Relatedness and Diversity of *Staphylococcus aureus* from Different Reservoirs: Humans and Animals of Livestock, Poultry, Zoo, and Aquaculture

**DOI:** 10.3390/microorganisms8091345

**Published:** 2020-09-03

**Authors:** Vanessa Salgueiro, Vera Manageiro, Narcisa M. Bandarra, Eugénia Ferreira, Lurdes Clemente, Manuela Caniça

**Affiliations:** 1 National Reference Laboratory of Antibiotic Resistances and Healthcare Associated Infections (NRL-AMR-HAI), Department of Infectious Diseases, National Institute of Health Dr. Ricardo Jorge, 1649-016 Lisbon, Portugal; vanessa.salgueiro@insa.min-saude.pt (V.S.); vera.manageiro@insa.min-saude.pt (V.M.); eugenia.ferreira@insa.min-saude.pt (E.F.); 2 Centre for the Studies of Animal Science, Institute of Agrarian and Agri-Food Sciences and Technologies, Oporto University, 4051-401 Oporto, Portugal; 3 Divisão de Aquacultura, Valorização e Bioprospeção, Departamento do Mar e Recursos Marinhos, Instituto Português do Mar e da Atmosfera, 1749-077 Lisboa, Portugal; narcisa@ipma.pt; 4 INIAV–Instituto Nacional de Investigação Agrária e Veterinária, 2780-157 Oeiras, Portugal; lurdes.clemente@iniav.pt

**Keywords:** MRSA, ST398, Portugal, animals, human isolates

## Abstract

The main aim of this study was the characterization of antibiotic resistance mechanisms in 82 *Staphylococcus aureus* strains isolated from humans and animals. Antibiotic susceptibility testing was performed on all *S. aureus* isolates accordingly, and antibiotic-resistant genes were investigated by genotypic methods. The genetic diversity of *S. aureus* was studied through *spa*, multilocus sequence typing (MLST), and *agr* typing methods. The majority of *S. aureus* from human sources were resistant to cefoxitin (and harbor the *mecA* gene) and fluoroquinolones, whereas only four strains of *S. aureus* from animal sources revealed resistance to ciprofloxacin. In the set of *S. aureus* isolated from humans, the most frequent *spa*, MLST, and *agr* group were t032, ST22, and I, respectively. In strains from animal origin the most common *spa*, MLST, and *agr* group found were t2383, ST398, and III/not typable, respectively. *S. aureus* from humans and animals were identified either in clonal complexes CC5, CC30, and CC398, suggesting that they have the same putative founder in their evolution. Considering the three CCs encompassing strains from human and animal reservoirs with different *spa*-types, we can hypothesize that this might reflect an adaptation to different phylogenetic lineages in those reservoirs (host species) probably associated to genetic diversification of pre-existing strains.

## 1. Introduction

*Staphylococcus aureus* has a great capacity of dissemination, acquisition of new antibiotic resistances and production a variety of virulence factors, such as toxins (responsible for food poisoning). These virulence factors are controlled by *agr* gene, a central transcription regulator that responds to cell density and can be divided in four specificity groups [1,2,3].

Methicillin resistance is commonly observed worldwide among *S. aureus* isolated in hospitals (Methicillin Resistance *S. aureus*, MRSA) [4]. This resistance is usually encoded by *mecA* gene, which is responsible for the resistance to all β-lactam antibiotics, except for the fifth-generation cephalosporins. The *mecA* gene encodes for PBP2a (Penicillin-Binding Protein 2a), with low affinity for β-lactam antibiotics. A *mecA* homolog gene, the *mecC* gene, which also encodes a modified PBP, has been described in *S. aureus* [2,5]. Vancomycin is usually the antibiotic chosen for the treatment of infections caused by MRSA [6]. However, strains of *S. aureus* resistant to vancomycin (VRSA) have already been described. VRSA, with high-level MICs, is mediated by a *vanA* gene cluster, which is transferred from vancomycin-resistant *Enterococcus*. However, *S. aureus* low-level vancomycin resistant strains, non-*vanA* mediated, are more common. hVISA (heterogeneous-Vancomycin-Intermediate *S. aureus*) are characterized by a vancomycin susceptible MIC (Minimum Inhibitory Concentration) when tested by routine methods but with a subpopulation of cells that can grow in the presence of ≥2 mg/L of vancomycin. This heterogeneous phenotype appears to be related to a thickening of the cell wall and an increase of unbound peptidoglycan precursors, which bind to glycopeptide antibiotics and prevent their interaction with the precursors located at the cell wall [7,8].

In addition to being a commensal and an opportunistic pathogen in humans [1,9], these bacteria can also colonize birds and fish, and be maintained in the environment (water, air, and manure) [10]. In the last decade, MRSA has emerged as a significant animal health problem worldwide, representing an important economic burden, mainly in cattle, poultry, and pigs [9]. Likewise, these bacteria have been found in fish and shrimp from aquaculture origin, raising additional food safety concerns [11]. Several studies have reported possible transmissions from animal to man or vice versa [12,13,14]. These transmissions can occur through direct contact with animals or their products [10].

In this study, we aimed to identify *S. aureus* strains isolated from humans and animals (from livestock, poultry, zoo, and aquaculture) and to characterize their antibiotic resistance against structurally unrelated antibiotics (β-lactam antibiotics, glycopeptides, and fluoroquinolones). The genetic relatedness and diversity of these bacteria within these two environments was also evaluated.

## 2. Materials and Methods

### 2.1. Bacterial Isolates

This study included 58 *S. aureus* isolated from humans (with community and nosocomial origins), randomly selected from the strain collection of the National Reference Laboratory of Antibiotic Resistances and Healthcare Associated Infections in Lisbon. Strains were isolated from pus (*n* = 10), blood/cerebrospinal fluid (*n* = 6), exudates (*n* = 9), urine (*n* = 2), respiratory secretions (*n* = 14), ascitic fluid (*n* = 2), and unknown samples (*n* = 15).

Twenty-four isolates of *S. aureus* were collected approximately in the same period (between 2008 and 2018) from animal sources (bird, *n* = 1; bovine, *n* = 1; dolphin, *n* = 1; duck, *n* = 2; goat, *n* = 2; ovine, *n* = 1; rabbit, *n* = 6; swine, *n* = 2; waterbuck, *n* = 1; and gilthead seabream from aquaculture, *n* = 7), at Instituto Nacional de Investigação Agrária e Veterinária and at Instituto Português do Mar e da Atmosfera. Results were compared with the *S. aureus* isolates from humans.

All strains were identified by VITEK 2 and amplification of the 16S rRNA gene, as previously described [15].

### 2.2. Antibiotic Susceptibility Testing

Antibiotic susceptibility testing was performed by disk diffusion and E-test^®^, according to EUCAST guidelines (http://www.eucast.org/clinical_breakpoints/). The antimicrobials tested were cefoxitin (FOX; 30 µg), ciprofloxacin (CIP; 5 µg), vancomycin (VA; 0.016–256 µg/mL), and teicoplanin (TP; 0.016–256 µg/mL) for all *S. aureus. S. aureus* strain ATCC 25,923 was used as a quality control.

The isolates were considered multidrug resistant if they presented resistance to three or more structurally unrelated antibiotics.

### 2.3. Glycopeptide Resistance Detection (GRD)

All strains resistant or with borderline breakpoints to vancomycin or teicoplanin were subjected to a GRD test (BioMérieux, Hazelwood, MO, USA), according to manufacturer’s instructions, for identification of hGISA (heterogeneous Glycopeptide-Intermediate *S. aureus*); the test was considered positive when the result was ≥8 µg/mL for either vancomycin or teicoplanin, and standard vancomycin MIC < 4 µg/mL.

### 2.4. DNA Extraction

DNA was extracted using MagNa Pure 96 Instrument (Roche, Basel, Switzerland), according to manufacturer’s instructions, or using lysostaphin as previously described [16].

### 2.5. Detection of mec Genes

*S. aureus* that demonstrated resistance to cefoxitin were investigated for the presence of *mecA* and *mecC* genes by multiplex PCR. A 23 µL final reaction mixture included: buffer (1×, Qiagen, Hilden, Germany), dNTPs (0.5 mM of dATP, dCTP, dGTP and dTTP, Roche Diagnostics, Basel, Switzerland), MgCl_2_ (3 mM, Qiagen), Q solution (1×, Qiagen), primers (0.4 µM; [17]), Taq polymerase (1 U, Qiagen), and sterile double distilled water. Two microliters of DNA were added to this final reaction mixture and the PCR reactions conditions were as follows: initial denaturation at 94 °C for 5 min, followed by 30 cycles of 94 °C for 30 s, 59 °C for 1 min, and 72 °C for 1 min with a final extension at 72 °C for 10 min.

### 2.6. spa Typing

*spa* typing was performed to all *S. aureus* using the aforementioned PCR reaction, using the primers described by others [18]. The PCR products (5 µL) were purified using illustra™ ExoProStar™ 1-Step and the following thermal cycling conditions: 37 °C for 15 min, followed by 80 °C for 15 min. The purified products were sequenced with the automatic sequencer ABI PRISM^®^ 3100 (Applied Biosystem, Foster City, CA, USA). The sequences were analyzed with the software BioNumerics© Applied Maths and the *spa* types were identified using the database available at http://spatyper.fortinbras.us/. New *spa* types were submitted and accepted in the database Ridom SpaServer (http://spaserver.ridom.de/).

### 2.7. Multilocus Sequence Typing (MLST)

The seven housekeeping genes (*arcC*, *aroE*, *glpF*, *pta*, *gmk*, *tpi,* and *yqiL*) were amplified for all *S. aureus* isolates, as already described [19]. The PCR products were purified, sequenced, and analyzed as described above. The database available at https://pubmlst.org/saureus/was consulted for the determination of the sequence type (ST) and submission of new ST.

### 2.8. agr Typing

The accessory gene regulator (*agr*) was also studied for all *S. aureus* isolates. The PCR reaction mixture had the same composition as that described above, using the primers described by others [20]). The thermal cycling conditions differed from those used for the *mec* genes only in the annealing temperature that was 59.8 °C for 30 s.

### 2.9. Minimum Spanning Tree

A minimum spanning tree (MST) was built based on eight genes (*arcC*, *aroE*, *glpF*, *pta*, *gmk*, *tpi*, *yqiL,* and *spa*) of all *S. aureus* isolates with the software PHYLOViZ Online (available at: https://online.phyloviz.net/index), which uses the goeBURST algorithm [21] and its expansion for representing the possible evolutionary relationships between strains.

## 3. Results

### 3.1. Phenotypic Analysis

The majority (69.0%) of *S. aureus* from human sources were resistant to cefoxitin (with diameter of inhibition ranging from 6 to 21 mm) which classifies them as MRSA, and also presented a high percentage of resistance to ciprofloxacin (81.0%; with diameter of inhibition zone from 6 to 20 mm). Furthermore, human strains also showed decreased susceptibility to teicoplanin (1.7%), with MIC of 6 µg/mL (Table 1). One *S. aureus* (1.7%) isolate was multidrug resistant and one strain was characterized as hGISA, with a positive result of ≥8 µg/mL for teicoplanin and standard vancomycin MIC of 2 µg/mL.

On the contrary, the 24 *S. aureus* isolated from animals revealed susceptibility profiles to almost all antibiotics tested, except for four strains recovered from rabbits, which showed resistance only to ciprofloxacin, with diameter of inhibition zone ranging from 6 to 10 mm (Table 1).

### 3.2. mec Genes in S. aureus

Of the 40 strains resistant to cefoxitin from human sources, 39 were positive for the *mecA* gene, thus confirming this phenotype. The exception was a cefoxitin resistant strain, which showed a negative result for both *mecA* and *mecC* genes. The *mecC* gene was not found in any of the *S. aureus* isolates.

### 3.3. Genetic Relatedness and Diversity of S. aureus

Regarding *agr*-typing, among all *S. aureus* isolated from humans, 31 (53.4%) belonged to group I, 19 (32.8%) to group II, and 3 (5.2%) to group III. No strains belonging to group IV were detected and five isolates were negative for the presence of any of the four groups. The search of *agr* gene in the *S. aureus* from animal sources revealed 3 (12.5%) strains belonging to group I, 3 (12.5%) to group II, 7 (29.2%) to group III, and 4 (16.7%) to group IV. Seven strains (29.2%) from aquaculture were *agr* non-typable.

Twenty-nine different *spa* types were identified for *S. aureus* from human origin and two types were here described for the first time (t14878 and t14933). For the strains from animal origin were detected 12 different types of *spa*, among which one new type (t15307). In the group of isolates from human sources, the most frequently identified type of *spa* was t032, whereas in the group isolated from animal sources the most abundant was t2383. The *spa* type t571 was found in both reservoirs.

**Figure 1:**
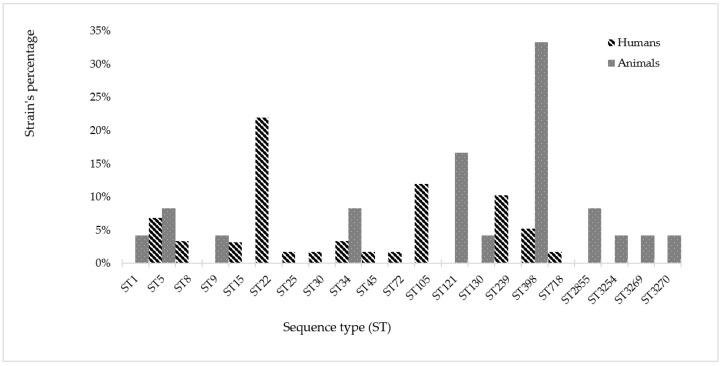
Distribution of *S. aureus* sequence typing (ST) clonal lineages of human and animal origin.

MLST revealed 13 different ST among the strains collected from human sources and 11 ST among the strains from animal sources (ST3254, ST3269, and ST3270 were here described for the first time). The ST5, ST34, and ST398 were found in both reservoirs. The most frequently identified ST in *S. aureus* from human origin was ST22, and ST398 was the most frequent in *S. aureus* from animal origin (Figure 1).

Table 2 shows the various genes studied for *S. aureus* bacteria and the respective phenotypes.

In isolates of human origin, the resistance to cefoxitin and ciprofloxacin were widely dispersed by the various *spa* types, ST, and *agr* groups. On the contrary, teicoplanin resistance was found only associated with *spa* t002, ST105, and *agr* group II. As for the new *spa* types, t14878 was associated to one strain susceptible to all antibiotics tested and t14933 was related with a cefoxitin and ciprofloxacin resistant strain. Furthermore, in the group of the isolates from humans, two strains whose *spa* gene could not be amplified belonged to ST105 and *agr* group II and revealed only resistance to ciprofloxacin.

In strains of animal origin, resistance to ciprofloxacin was associated with two *spa* types, t1190 and t645, two ST, ST2855 and ST121, and two *agr* groups, III and IV. The only new *spa* in this group, t15307, was associated with a new ST, ST3270, belonged to *agr* group I and was susceptible to all antibiotics tested. The other two new ST, ST3254, and ST3269, belonged to different *spa* types (t748 and t1166, respectively), different *agr* groups (III and I, respectively) and had susceptibility phenotypes to all antibiotics tested.

**Figure 2:**
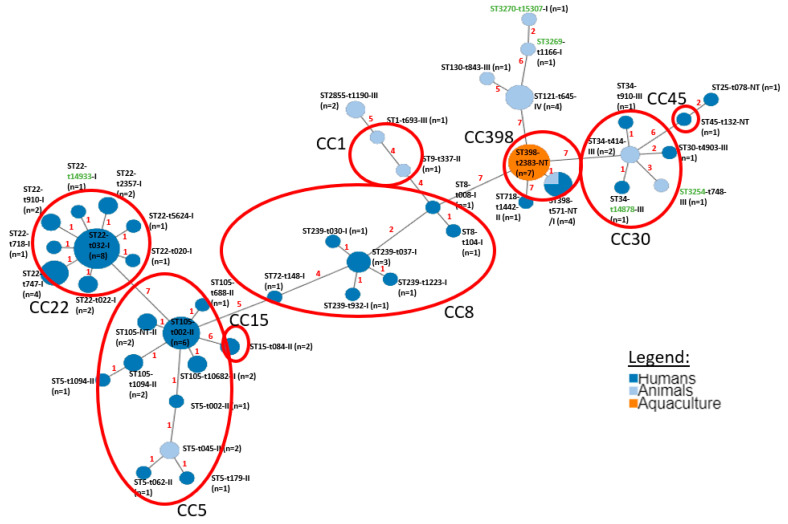
Minimum spanning tree based on the various *spa*, ST, *agr,* and clonal complexes (CC) types found in different reservoirs, in this study (humans, animals, and aquaculture; here aquaculture is in a different color from the other animals, to highlight that all of these strains belong to ST398 and *spa* type t2383). ST and *spa* types in green represent those described here for the first time. CC are shown by red circles. Numbers in red corresponds to the link length, which is proportional to the number of differences by its scalability (Scaling Factor: 7).

The minimum spanning tree based on the *spa*-types and ST found in this study generated allelic profiles and their associated data, including *agr*-typing (Figure 2). It shows that both clonal complexes CC5 and CC30 grouped strains from animal and human origins. Furthermore, the seven ST398 strains from aquaculture origin differed from the remaining four (one from animal and three from human origin) by *spa*-type.

## 4. Discussion

Antibiotic resistance is a complex phenomenon involving several resistance mechanisms and affects different bacterial species and genera in the most diverse environments, from hospitals to communities and animals, and thus becoming a growing public health problem. Given this scenario, it is indispensable to monitor and collect information on resistance genes in the various reservoirs, and to try to contain or even prevent eventual dissemination; this is the reason that led to the elaboration of this study.

The high prevalence of cefoxitin and ciprofloxacin resistances in human strains (Table 1) agrees with other Portuguese studies [22], as well as other countries [23,24]. On the contrary, countries like Gambia have much lower rates [25]. Of the 40 strains resistant to cefoxitin, 39 had the *mecA* gene and only one was negative for both *mecA* and *mecC*. In this last case, cefoxitin resistance may be due to a high yield of a penicillinase capable of slowly degrading these antibiotics or to changes in the genes coding for PBP, leading to amino acid substitutions in the transpeptidase domain [26]. An alternative explanation is related to mutations in the hybridization zone of the primers that prevented the amplification of these genes.

In the present work, no MRSA were found among strains from animals, but other studies have already described them, namely in pigs [27] and calves [28] in Portugal, in wild animals such as deer, goats, vultures, and wild boars in Spain [29] and in sheep and goats, as well as their milk, in Nigeria [30]. These cases, although MRSA prevalence rates have been low, are a concern not only for animal health but also for human health since these animals can serve as reservoirs of resistance genes that can be disseminated among different settings.

In relation to infections caused by MRSA and in patients unable to tolerate vancomycin, fluoroquinolones (that belong to the third most commonly antibiotic class prescribed in Portugal [31]) have become one of the treatment options [22,32]. The resistance mechanisms to ciprofloxacin were not the aim of this study but others indicate that in *S. aureus* the primary target is topoisomerase IV (more specifically mutations in the *parC* gene), which normally leads to moderate levels of resistance. Usually, these mutations precede other mutations in *gyrA* gene. Another resistance mechanism to this class of antibiotics in *S. aureus* is the expression of a chromosome encoded MFS (Major Facilitator Superfamily) family efflux pump, NorA, which has the ability of extrusion these antibiotics; thus, conferring a low-level resistance [32]. Indeed, our strains showed a large range of diameter of inhibition zone for resistance fluoroquinolones (from 6 to 20 mm), which might be related with both of those mechanisms, singly or in association.

Teicoplanin is often used in cases of septic arthritis and osteomyelitis caused by MRSA. The resistance to teicoplanin found in the present study may be related with the presence of *tca*RAB (a teicoplanin resistance operon) or the inactivation of *tca*A. This strain is also hGISA, thus probably registering alterations in the cell wall, as already described here. These heterogeneous populations represent a clinical challenge since they can lead to treatment failure. Sometimes, cross-resistance between teicoplanin and vancomycin can be observed [33].

In the group of strains isolated from humans, t032 (ST22) was the most frequent *spa* type followed closely by t002 (ST5/ST105). These findings agree with the global frequencies of these *spa* types (10.10% for t032 and 6.59% for t002; http://www.spaserver.ridom.de/) and Portuguese studies in the community [34] and in hospitals [22]. Portuguese hospitals have so far described several stages in the clonal dissemination of MRSA: first, in 1992 and 1993, the Iberian clone ST247-t008/t051 replaced the Portuguese clone ST239-t421. In 1994 and 1995, occurred the rapid spread of the multidrug resistant Brazilian clone ST239-t037 (found in three strains collected in hospitals, in our study); later, this clone was replaced by the ST22-t032 epidemic clone (in eight strains from our study). Shortly afterwards, the New Yorker/Japanese clone ST5-t067 and, more recently, the ST105-t002 clone appeared as the second most prevalent clones [35] (which was found in five strains from our study). For the other clones in this study, the ST-*spa* association had already been described for several cases: the clone ST8-t008 in humans and pigs in Norway; the ST22-t020 and the ST5-t179 in the community in Portugal; the ST239-t932 in Malaysia; the ST5-t062 in Brazil; the CC45-t132 and CC25-t078 in Lebanon; the ST239-t030 in China; the ST8-t104 in Angola; the ST15-t084 in Iran associated with hospitals; the ST22-t022 in USA, Canada, Europe, Middle East, Asia, and Australia/New Zealand; the CC5-t688 in USA associated with the community; ST22-t747/t910/t2357 and CC5-t10682 in Portugal associated with hospitals and the community; the CC5-t1094 in rabbits in Portugal; and the ST72-t148 in humans, gorillas, and chimpanzees in Gabon [2,4,22,34,36,37,38,39,40,41,42,43,44,45]. Community-based strains used to be associated with ST1, ST8, ST30, ST72, and ST80; however, except for ST1 and ST80, the other ST were found in this study in hospital-acquired strains. In fact, a 2013 study in Portugal showed that barriers between hospital and community are becoming smaller and the ST most frequently found in the community are also identified in hospitals, especially ST22 and ST5/105 [46].

In *S. aureus* strains isolated from animals, there were some ST-*spa* associations previously described, such as the ST5-t045 clone in dogs and cats in the USA [47], although in this study it was found in goats; the ST9-t337 in pigs in Thailand [48], as in the strains studied here; the ST398-t571 in pigs and humans in Korea and the USA, respectively [49,50], having been found in a dolphin and humans in our study; the CC398-t2383 in pigs and humans from Denmark and Netherlands, respectively [51,52], while in this study this clone was found in all strains from gilthead seabream from aquaculture origin; the CC130-t843 in humans in the United Kingdom, France, and Spain, and in dogs in the United Kingdom and Germany, in cattle in the United Kingdom, Denmark, and France, among others [53], having been found here in a bird of a zoo; CC133-t1166 in a goat in Tunisia [54], having been identified in a duck in our sample; and ST2855-t1190 in hares [55], associated with rabbits in this study. The exceptions were the clone ST3270-t15307, because it was described here for the first time associated with a duck, and the *spa* type t748 (collected in a pig) associated with a new ST, the ST3254, which were previously associated with ST239 in hospitals in China [40]. In the present study, in *S. aureus* from animals we identified three important clones known to cause infections in humans and animals, ST398-t571/t2383 and ST130-t843. Effectively, ST398 is one of the most worrying ST, since it become a rapidly emerging cause of human infections, most often associated with livestock exposure. In Portugal, CC398 has already been identified in MRSA strains responsible for community acquired infections [46], and animals such as in breeding calves [28], companion animals [56], wild animals [16], and among healthy pigs [57]. It is not only the MRSA isolates from this clone that cause important infections, because cases of septicemia and infections of the skin and soft tissues caused by methicillin-susceptible *S. aureus* (MSSA) belonging to ST398 have also already been registered [58]. All seven *S. aureus* from gilthead seabream belonged to ST398; to our knowledge, this is the first description of ST398 in fish from aquaculture, being mostly described in livestock, poultry and wildlife [10], and as associated to a high virulence [59] is of concern.

In this study, the resistance to glycopeptides (hGISA strain included) was exclusively associated with clone ST105-t002-*agr* type II, as in previous studies, like Sakoulas et al. [60], Moise-Broder et al. [61], and Purrello et al. [62]. The mechanisms by which this happens are still unclear, but there seems to be a selective advantage of some clones associated with *agr* group II towards a selective pressure by the presence of glycopeptides [61].

For two strains isolated in a hospital, it was not possible to amplify the XR region of protein A encoded by the *spa* gene. This has already been described and may be due to a complete absence of the *spa* gene or to deletions/insertions in the region encoding the IgG binding domain of the protein A. This region is upstream of the XR region where it hybridizes the primer forward; thus, preventing the amplification.

The *agr* locus acts in the presence of a high extracellular concentration of the called Autoinducing Peptide (AIP), this concentration being proportional to the bacterial population density. Interestingly, AIP segregated by a group, can inhibit the expression of an *agr* from a different group, which may lead to cooperation between strains belonging to the same *agr* group and competition between strains with different *agr* groups. Strains of *S. aureus* belonging to the same *agr* group are thus considered to have similar biological properties and a close genetic relationship [60,62]. In strains collected in humans, group IV was not found and group I was the most prevalent, as in a study by Azmi et al. [63]. A distinct distribution was observed in strains collected from animals, since here the predominant group was III, unlike other studies such as Smyth et al. [64], and there were representatives of all four groups in this samples. Other studies have demonstrated the predominance of group III, but in strains of MRSA from deer [65], sheep [66], and rabbit [38].

The MST analysis (Figure 2) shows that CC5 (ST5 and ST105) and CC30 (ST30, ST34, and ST3254) grouped strains from both animal and human origins. In CC5, ST5 strains from human and animal origin have the same putative founder ST105-t002-II. Lowder et al. have suggested a recent human-to-poultry host jump of this lineage, favored by a close contact between these two hosts, showing that ST5 is a well-adapted lineage to human hosts, often related with community- and hospital-associated MRSA [67]; in the adaptation to this recent host (poultry), this lineage lost the function of several genes implicated in pathogenesis of human infections and acquired mobile genetic elements (MGE). This lineage is known for a great capacity of acquiring MGE which contributes to the adaption to new hosts like poultry and pigs [67,68]. The eventual transmission between reservoirs (e.g., human–animal–human) might be confirmed by genomic approaches. Furthermore, as belonging to the same *agr* group II, it might also be considered that ST5 strains have related biological properties and a close genetic relationship. Regarding CC30, the ST30 and its single locus variant (SLV) ST34 from human origin, and the new ST3254 from animal origin (double locus variant of ST30), might have the same founder ST34-t414-III, from animal origin; all belong to the *agr* group III. The seven ST398 strains from aquaculture origin had different *spa*-types and *agr* groups (mostly non-typable or from group I) when compared with the other ST398 strains (one from animal and three from human origin). Considering the three CCs encompassing strains from human and animal reservoirs with different *spa*-types, we can hypothesize that this might reflect an adaptation to different phylogenetic lineages in those reservoirs (host species) and are eventually associated to genetic diversification of pre-existing strains, which have consequences in the spread control of these strains between reservoirs.

## 5. Conclusions

Overall, in this study we evaluated the susceptibility to four antibiotics belonging in three different classes of 82 *S. aureus* strains collected in humans and animals and evaluated the genetic relatedness and diversity of these bacteria within several environments.

In *S. aureus* from human origin, resistance to fluoroquinolones and cefoxitin predominated, the latter caused by expression of the *mecA* gene. Portugal, despite a decreased of MRSA in the years of 2015–2018, still has the highest rates among European Union/European Economic Area countries [69]. Effective strategies are needed not only in hospitals, but also in health care facilities and veterinary institutions to reduce the dissemination of this bacterium, which is a public health problem today. Reduced susceptibility to teicoplanin was also registered, which although poorly disseminated are of concern, namely after the first identification of VRSA (Vancomycin-Resistant *S. aureus*) in Europe, in a Portuguese hospital [70]. Two new *spa* types (t14878 and t14933) were found in *S. aureus* strains from humans and one new *spa* type (t15307) and three new ST (ST3254, ST3269, and ST3270) in strains collected from animals. Types of *spa* and ST found in both humans and animals demonstrate the spread of clones between different reservoirs. We highlight the first description of ST398 in fish from aquaculture. These findings, together with reports of MRSA CC398 from livestock, healthcare-associated or hospital-acquired MRSA, and community-associated MRSA [67,71], show that CC398 is being increasingly disseminated in MSSA and in other animal reservoirs than livestock, such as in aquaculture. It is increasingly important to distinguish between the different reservoir-adapted clades of MSSA and of MRSA to better control the spread paths and to implement more focused measures. High throughput sequencing (such as whole genome sequencing) will be helpful to elucidate eventual re-adaptation of MSSA and/or MRSA to different reservoirs and if transmission is human–animal–human or other, as in the case of ST5 from CC5 (Figure 2). This study highlights the increasing importance to control the spread of *S. aureus* between several reservoirs.

## Figures and Tables

**Table 1:** Antibiotic susceptibility results from *S. aureus* isolated from humans and animals.

Antibiotic	S. *aureus* No. (%)			
	Humans (*n* = 58)		Animals (*n* = 24)	
	R	S	R	S
FOX	40 (69.0)	18 (31.0)	0 (0.0)	24 (100.0)
CIP	47 (81.0)	11 (19.0)	4 (16.7)	20 (83.3)
TP	1 (1.7)	57 (98.3)	0 (0.0)	24 (100.0)
VA	0 (0.0)	58 (100.0)	0 (0.0)	24 (100.0)

FOX, cefoxitin; CIP, ciprofloxacin; VA, vancomycin; TC, teicoplanin.

**Table 2:** Association between the genotype and the phenotype of the 82 strains of *S. aureus* from two different reservoirs (humans and animals).

*spa* Type	MLST	*agr* Group	mecA	Resistance Profile
**S. *aureus* from humans (*n* = 58):**				
t002 (*n* = 6)	ST5, ST105	II	+	FOX, CIP, (TP)
t008 (*n* = 1)	ST8	I	+	FOX, CIP
t020 (*n* = 1)	ST22	I	+	FOX, CIP
t022 (*n* = 2)	ST22	I	+	FOX, CIP
t030 (*n* = 1)	ST239	I	-	CIP
t032 (*n* = 8)	ST22	I	+	FOX, CIP
t037 (*n* = 3)	ST239	I	+	FOX, CIP
t062 (*n* = 1)	ST5	II	-	-
t078 (*n* = 1)	ST25	NT	-	-
t084 (*n* = 2)	ST15	II	-	(FOX), CIP
t104 (*n* = 1)	ST8	I	+	FOX, CIP
t132 (*n* = 1)	ST45	NT	-	-
t148 (*n* = 1)	ST72	I	-	-
t179 (*n* = 1)	ST5	II	+	FOX, CIP
t571 (*n* = 3)	ST398	NT	-	-
t688 (*n* = 1)	ST105	II	-	CIP
t718 (*n* = 1)	ST22	I	+	FOX, CIP
t747 (*n* = 4)	ST22	I	+/-	(FOX), CIP
t910 (*n* = 3)	ST22, ST34	I, III	+/-	(FOX), (CIP)
t932 (*n* = 1)	ST239	I	+	FOX, CIP
t1094 (*n* = 3)	ST5, ST105	II	+	FOX, CIP
t1223 (*n* = 1)	ST239	I	-	CIP
t1442 (*n* = 1)	ST718	II	-	-
t2357 (*n* = 2)	ST22	I	+	FOX, CIP
t4903 (*n* = 1)	ST30	III	-	-
t5624 (*n* = 1)	ST22	I	+	FOX, CIP
t10682 (*n* = 2)	ST105	II	+	FOX, CIP
t14878 ^1^ (*n* = 1)	ST34	III	-	-
t14933 ^1^ (*n* = 1)	ST22	I	+	FOX, CIP
NT (*n* = 2)	ST105	II	-	CIP
**S. *aureus* from animals (*n* = 24):**				
t045 (*n* = 2)	ST5	II	-	-
t337 (*n* = 1)	ST9	II	-	-
t414 (*n* = 2)	ST34	III	-	-
t571 (*n* = 1)	ST398	I	-	-
t645 (*n* = 4)	ST121	IV	-	(CIP)
t693 (*n* = 1)	ST1	III	-	-
t748 (*n* = 1)	ST3254 ^2^	III	-	-
t843 (*n* = 1)	ST130	III	-	-
t1166 (*n* = 1)	ST3269 ^2^	I	-	-
t1190 (*n* = 2)	ST2855	III	-	CIP
t2383 (*n* = 7)	ST398	NT	-	-
t15307 ^1^ (*n* = 1)	ST3270 ^2^	I	-	-

^1^*spa* type first identified in this study; ^2^ ST first identified in this study; NT: not typable; - Susceptible to all antibiotics studied; and variable presence of nonsusceptibility phenotype is indicated by parentheses.
